# Abstracts and Gleanings

**Published:** 1881-08-20

**Authors:** 


### ABSTRACTS AND GLEANINGS.

**Post Partum Hemorrhage.**—Dr. Thomas, in Society of the County of Kings, says: The particular point which I wish to make is this: In ninety-nine out of one hundred cases of post-partum hemorrhage, seen before the woman's nervous system is entirely prostrated, if the hand is properly introduced to the fundus of the uterus, everything turned, and the walls of the organ irritated with the pulps of the fingers, firm uterine contraction will take place. I do not believe there is any remedy to be compared with it, any more than I believe there is any remedy in the treatment of malarial fever to be compared with quinine.

But let us suppose that in the one hundreth case it fails to accomplish this result. Then it is said there are other agents which stimulate the uterus more than the hand. I do not believe a word of it. I believe there are things which have stimulated the uterus to contraction where the hand has failed; but I believe also it was the hand of a man who has not used it properly.

With reference to the injection of a solution of iron, as recommended by Dr. Barnes, I reject the measure entirely and absolutely, except as a dernier resort. Hot water may be injected to the fundus with perfect safety, or put there in a sponge, and it will be very likely to stimulate the uterus; but not nearly so well as the hand. How did Dr. Stuart's sponge effect the result which he mentioned? I do not believe that it was the hot water which it contained, but the sponge itself, which as a foreign body, was brought in contact with lax fibres of the uterine wall, and stimulated them to contraction. Another substance which has been used is the tincture of iodine; and still another, which I regard as better than iodine, and which has been highly recommended by Dr. Penrose, of Philadelphia, is common vinegar. One advantage which vinegar possesses is, that it can be obtained in any house. It may be carried in upon an ordinary handkerchief, or bit of soft cloth, swept over the walls of the uterus, and two things are accomplished: 1. The introduction of a foreign body causes the uterine fibre to litigate the bleeding vessels; and 2. The vinegar probably has some influence in causing coagulation of blood. But I am not sure that it is not the hand even then which accomplishes the end desired.

In the hypodermic use of ergot we have a most valuable measure to aid us in securing uterine contraction. To give this remedy by the rectum is hardly practicable, and if administered by it the woman will almost certainly vomit at once; and besides, if it remains in the stomach, I doubt if it is absorbed under such circumsiances.

The hypodermic injection of 15, 20 or 30 drops of sulphuric ether stimulates the nervous system, and in that way arouses the uterus to accomplish its work.

But there is one remedy which has been overlooked. In works on obstetrics it is scarcely mentioned; and it has received very little mention in periodical literature. It is the Faradic current applied directly to the uterine wall. I have employed it in only one case, but others have used it successfully, and in my case it was either a coincidence or an extraordinary result. Almost everything had been used except Dr. Barnes' method, and without permanent result; but when I took one flat sponge electrode and carried it into the uterus, and placed the other upon the nape of the patient's neck, and allowed a strong current to pass, firm uterine contraction immediately ensued, which seemed to save the woman's life. Although this is but a single case, I think the remedy is worthy of a fair trial. The question arises, Why has it not been tried before? Chiefly because a battery was not at hand and could not be obtained, and when obtained, as a rule, it would not work, never under any circumstances. But those days have passed. Nearly every physician has a battery now; and a good battery can be obtained much more readily anywhere than good ergot, except in Brooklyn.

Now, in conclusion, Mr. President, I will say that in the treatment of post-partum hemorrhage the rule should be this:

If the hemorrhage is slight, and for good reasons you do not wish to pass the hand into the uterine cavity, try the hypodermic use of ergot; apply excessive cold or excessive heat to the fundus, force the uterus into firm contraction under your hand, and never let go of it until the woman stops bleeding. How long shall you hold the uterus? I have repeatedly held it, under such circumstances, for twelve hours.

But suppose it fails and the hemorrhage continues. Then wash the hand and arm thoroughly with soap and water, use a nail brush thoroughly, dip the hand and arm in warm, strong carbolized water, and, without wiping them, carry the hand up to the fundus uteri, sweep everything out, and keep the hand there until the uterus contracts. Pass the pulp of the fingers up and down the sides of the uterus in any direction, and at the same time make counter-pressure from the outside with the other hand upon the wall of the abdomen.

If you fail with this, what next? It is a bad case, and you may resort to anything that produces a decided shock to the nervous system; give of ergot, brandy and ether hypodermically, and, lastly, give a fair trial to the Faradic current.

**The Summer Dysentery of Children.**—W. W. S. Watson, M. D., of New Holland, Illinois, in Michigan Medical News, says:

There are two forms of dysentery recognized by the authorities: the sporadic and the epidemic. It is essentially a disease of hot weather or hot climates, and the causes of its epidemicity is but an intensification or a prolongation of those which give rise to sporadic cases. The evidence leaves little room for doubt that the prime cause is a specific poison which is fanned into life by heat and other peculiar atmospheric conditions. What that poison is remains yet to be demonstrated. Post-mortem examinations of dysenteric subjects show the mucous lining of the colon and rectum to be the seat of the action of this morbific principle. The pathological condition is essentially an inflammation, in the progress of which mucus is freely thrown off; this inflammation may go on to ulceration, when the discharge becomes sanious and purulent. The fact of the affection being confined to the lower portion of the alimentary canal implies some disturbance of the portal circulation, which disturbance may bear the relation of cause or effect. The fact is that the portal circulation is obstructed in all or nearly all cases of dysentery, and success in treatment will very largely depend on the attention given to the restoration of the proper hepatic function.

Before indicating the more distinctive therapeutic agents employed by me, I would insist upon the importance of bodily rest to the patient. Any plan of treatment conducted without a regard to this precaution will prove comparatively futile. It is impossible to properly control the discharge and at the same time allow the patient to move about. Absolute rest in the recumbent position should be insisted on as a *sine qua non* of treatment. This is perhaps especially the case in the treatment of the disease as it affects children: with their delicate organization, the failure of treatment in many instances is probably due to the jolting to which the fond mother or sympathetic nurse subjects the fretful infant. The child should not be held even on the lap, but should be laid to rest on a pillow, and even in the removal of its diapers great care should be exercised that the child be not unnecessarily disturbed. Good ventilation should also be secured and all such articles of diet avoided as are liable to reach the inflamed tract undigested.

*Treatment.*—The first indication is to thoroughly rid the alimentary canal of irritating ingestia. This is most promptly effected by means of a saline purge, which, in addition to its action in this direction, also depletes the inflamed tissue through the exosmosis of serum which it causes. Castor oil is preferred by some; it is blander in its action but adds nothing to its purgative action. After this thorough cleansing of the canal the indication is to check the discharge. For this purpose I rely on opium and acids, after the following formula, modified, of course, according to age and other circumstances:

℞ Syr. rhei ........ ℥ jss

Spts. ammoniæ arom ........ ʒ ss

Spts. camphor ........ ʒ ss

Tr. opii ........ ʒ j

M. Sig. A teaspoonful every three hours to a child eight years old.

When there is much nausea a powder composed as follows may be given every alternate hour and a half:

℞ Bismuthi subnit ........ ℈ ij

Pulv. ipecac ........ ℈ ss

M. Div. in partes no. viij.

Camphor has a very marked controlling influence over this affection, and its combination with opium will be found invaluable.

In regard to the liver I would state that, notwithstanding the more recent views regarding the chologogue action of the mercurials, I still place my reliance on calomel. I find that when the discharge is largely composed of white mucus, destitute of a trace of bile, the administration of small doses of this preparation restores the deficient element, and with this restoration improvement in the condition of the patient follows. I do not wish to gainsay the physiological observations of the eminent experimenters who tell us that mercury is not a chologogue; all I do say, and I say it as the outcome of a considerable experience, is that as a therapeutic agent in the conditions to which I refer, it restores bile and is an important aid to the cure of dysentery. And further this deponent saith not.

**The Easy Administration of Medicines.**—Dr. G. F. Meeser, in Virginia Medical Monthly, says: The "easy administration of medicine" is a subject requiring the careful and thoughtful attention of physicians, as well as of interest for the convenience and pleasure of the patient. The advancement of homœopathy in certain sections of the country has depended, to a great extent, upon the easy and pleasant doses administered by followers of that school. The elegant pharmaceutical preparations compounded by the tasty and skilled chemist and pharmacist have done much to rob the physician's prescription of its terror and render the medicine palatable to the delicate patient.

Very recently, a new and important class of medicines has been introduced by the ingenious and enterprising house of Messrs. William R. Warner & Co., of this city, denominated "parvules." They bear evidence of exquisite taste and skill, and I have seen nothing of late which seems to supply a necessity so perfectly as they do.

The list of "parvules" prepared and kept in stock by this house comprises thirty-eight varieties. These "parvules" are, for the most part, composed of simple substances in minute globular form, less in sizes than granules, and are sold in small vials suitable for pocket cases. They are convenient, portable and easy of administration. The giving of small doses at short intervals, say every hour instead of every two or three hours, or three times a day, produces a more salutary effect.

The question of the ready solubility of these "parvules" claimed my attention, and this I proved to my perfect satisfaction by placing those containing camphor, etc., in the mouth, and then observing the effect.

I have seen nothing to please me more than these ready-prepared doses. I can give four parvules of aloin, each containing one-tenth grain, at bed time or at any time throughout the day, and get the full purgative effect desired without nausea or pain. I give these one at a dose, three times *daily* or *occasiondlly*, for habitual constipation, with the utmost benefit. When liver troubles also occur, I give parvules of podophyllin, each containing one-fortieth grain, in a similar manner. Two parvules of calomel, each containing one twentieth grain, given every hour for five doses, produces bilious evacuations, equal to ten grains of calomel as ordinarily administered.

These doses are in no sense homœopathic when given by this rule: One every hour, two every two hours, or three every three hours. To illustrate the fact that these are allopathic, let us take a parvule of morphia, one-fiftieth grain, and give one every hour. This would equal about one-half grain during the twenty-four hours The various medicated waters which are now so liberally patronized for their aperient effect, etc., I believe are harmful, because they disturb digestion by diluting the gastric juices, and reduce the temperature of the stomach. Parvules of aloin, one-tenth grain each, or of podophyllin, one-fortieth grain each, would replace the use of such waters most advantageously.

**A New Anæsthetic.**—In the Chemical News of April 2d, 1880, Mr. W. Bowman Maclene is reported to have introduced a new anæsthetic to the members of the Odonto-Chirurgical Society. This new nerve-stiller is a combination of ethylen-dichloride and ordinary nitrous oxide. The ethylen-dichloride is placed on a sponge in the tube through which the nitrous oxide passes. Only a small quantity of the former is required. Anæsthesia results in from one and a half to two and a half minutes. The sensation is said to be more profound and agreeable than when nitrous oxide is used alone. Mr. Maclene had used the anæsthesia sixteen times without any unpleasant resulting symptoms. In only one case was there stertor. The pulse was slightly accelerated but strong, while there was a complete absence of lividity, so repulsive to the looker-on. The muscular system also, instead of the rigidity characteristic of anæsthesia from nitrous oxide, was quite relaxed—thus greatly facilitating the manipulations of the operator. The Society resolved to experiment with this mixture.

Ethylen dichloride is composed of carbon, hydrogen and chlorine in the following proportions: $C_2 H_4 Chl_2$. It is a colorless liquid, having specific gravity of 1,271 at a temperature of zero (Centigrade); it has a pleasant, ethereal odor, and a sweetish taste; it boils at 85.5° (C). It is easily soluble in ether and alcohol, but insoluble in water. Passed through a red hot tube it is decomposed into carbon, hyrochloric acid and carbon dihydrate. It burns with a bluish-green flame. A solution of potassium hydrate in alcohol converts it into ethylen-monochloride by abstracting hydrochloric acid. Ethylen-dichloride is obtained by the direct action of chlorine on olefiant gas, and also by treating alcohol with chloride of phosphorus.—*Chicago Medical Gazette.*

**Hemorrhage During Labor.**—A. S. Clarke, M.D., in Proceedings of the Medical Society of the County of Kings, says: Naturally enough, hemorrhage during labor is much less frequent that at any other portion of the period of gestation, but it is not, for that reason, any less important. It can only arise from premature separation of the placenta, or some accident involving the maternal soft parts (independently of placenta prævia, the consideration of which is made a separate matter in the discussion of this evening).

The so-called accidental hemorrhage furnishes the largest proportion of cases of hemorrhage during labor. It arises from detachment of a normally situated placenta, and is rather more liable to occur before labor has begun, than during it. At any time in the last three months of utero-gestation this complication may be met with, as during that time the relation of uterus and placenta is changing, the adhesion being gradually weakened. If, from any cause, while this is going on, a small portion of placenta becomes separated from the uterine walls, effusion of blood is the result, and, as it increases in quantity, causes mechanically still more separation, and opportunity for the escape of still larger quantities. If the separation extends far enough to loosen the placental margin, the blood will escape between membranes and uterine walls, and so make known the nature of the accident. But more frequently the margin remains intact, and the blood is pent up between placenta and uterus, and only makes its presence known by the constitutional symptoms which, fortunately, are generally pretty direct and easily made out, if the hemorrhage is excessive. It is needless for me to go into details in this matter, as my duty is simply to outline the subject, leaving causes, symptoms and treatment for the gentleman who will discuss it later in the evening. Of course the only remedy is in securing prompt and efficient uterine contraction, and just here comes to my mind the principal difficulty. This accident generally occurs in women of feeble constitution, or relaxed fibre from repeated child-bearing, or from some abnormal condition of blood—hence they are but ill-able to bear the shock of serious operation. All authorities agree that the uterus should be emptied of its contents as speedily as consistent with the condition of the patient, but undue haste may increase the collapse, and cause instant death. Dilatation of the cervix, by Barnes' method (after rupture of the membranes, which should be done at the outset), to be followed, as soon as may be with safety, either by version (the bi-polar method if possible), or the forceps if the head is low down. It is often difficult to secure firm contraction, owing to the condition of the uterus, and authorities differ in their suggestions in this regard. Dr. Barnes recommends strongly the application of a styptic to the entire cavity, and kneading and compressing the uterus to control hemorrhage. Ergot is used by some, and objected to by others, on account of its depressing effect on an already depressed patient. These points of treatment, I hope, will be brought out in the discussion this evening, that we may hear what methods have given the best results in the hands of gentlemen who have had experience in these cases. I doubt if the styptic plan is one which meets with much favor, except under circumstances where all else is likely to fail.

**Thumb-Sucking**.—Dr. D. H. Goodwillie, of New York city, at American Medical Association, reported a case and illustrated it by a wax model. The treatment consisted in breaking up the habit by applying a leather pad to the elbow, preventing the hand from coming to the mouth; and nasal catarrh is to be treated by douches and the application of powder blown into the nose, proper food, clothing and rest. His conclusions were as follows:

1. Thumb-sucking is more disastrous to the health of the child than the sucking of the other fingers; for the thumb, once in the mouth, it more readily remains during sleep.
2. It interferes with the child's proper rest, which should be continuous and undisturbed, and so becomes a source of nervous irritation and exhaustion.
3. It interferes with the natural respiration through the nose, and sets up abnormal conditions.
4. It malforms the anterior part of the mouth and affects proper mastication.—*Va. Med. Monthly.*

**Phosphide of Zinc in Locomotor Ataxy.**—Two cases of ataxy are reported by Dr. Hastings Burroughs, of Paris, in which very great benefit was obtained by the use of phosphide of zinc. The drug was given in doses of one-tenth of a grain per day, increased to half a grain per day.—*Medical Press and Circular.*

**Adherent Placenta.**—I have met with several cases of morbidly adherent placenta, during the last fourteen years, and am inclined to believe that the diagnostic problem be solved with almost absolute certainty; although from my experience being limited to so short a time, I would desire to write with all becoming modesty.

The diagnosis is, I think, to be founded upon two symptoms; one of which is mentioned by Dr. Churchill, the other by Dr. Barnes, viz: that at some period of pregnancy, generally between the third and fifth month, a fixed pain, generally of a dull aching character, is felt over some part of the uterus; and this is converted into a severe *dragging* pain when the patient attempts to turn over to lie on the side opposite to the placental side; so much so that patients, with an adherent placenta, will never (as far as my experience goes,) voluntarily lie on that side. This pain I believe to be of the same nature as that mentioned by Dr. Barnes, as being experienced when the cord is drawn upon; and is due to the dragging on the cord by the child, when from gravitation it sinks through the liquor amnii.

Theoretically, it may be objected to this explanation that usually the cord is sufficiently long to prevent any such dragging; but I think it will generally be found that when the cord is long, it is twisted around the neck or limbs of the child, and produces the same effect as a short cord would.

No history of this dragging pain on the patient's turning to the side opposite to the placental insertion will be obtained, when the retention of the after-birth is merely due either to the inertia of a wearied uterus, or from irregular contraction; if there is hemorrhage in either of these cases, one would be justified in trying the effect of cold, compression, etc., before introducing the hand, but in cases of true placental adhesion, trying these and similar means lead to dangerous loss of time.—*Obstetric Gazette.*

**Enlarged Tonsils.**—Dr. Pancoast says, in Clinical Record: I examined this little boy a few days ago, and found his tonsils much enlarged, reaching just beyond the level of the pillars of the fauces. I recommended and told him to come here to-day tor operation, by partial excision, which I thought might be necessary, if it persisted. I find now, upon examining him, that he does not need the operation. I now see that the glands have subsided and returned to the level of the half arches. When at all large it is necessary to remove part of the tonsil, because it acts mechanically like a foreign body, by interfering with breathing and keeping up irritation. The antiphlogistic effect of the operation of partial excision makes the gland shrink to its normal size.

In the early stage you can often remove the swelling and relieve the patient, by a procedure which I call the "therapeutic touch of the antiphlogistic knife;" multiple punctures in the swollen tonsil, made deep enough to relieve the congestion and exudation, and empty the overfilled vessels, so as to produce an antiphlogistic effect. This I did the other day, when I saw him first. The result has been quite satisfactory. It is also proper in such cases to give a good gargle, and tonics internally.

**Epigastric Pressure in Obstinate Hiccough**.—The *Jonrnal des Sciences Medicales de Louvain*, relates that M. Deghilage, of Mons, was called to a young lady suffering from very violent hiccough, with spasm of the glottis. The patient had been over an hour in this state, and was unable to articulate a syllable. There was no fever, no signs of heart trouble ; the only cause that could be assigned was that the patient had the lower limb chilled a few days previously, during her menstrual period. Inhalation of vinegar and Hoffman's anodyne, and the application of sinapisms had been tried without effect. Recalling Rostan's precept for such cases, M. Deghilage applied the palm of the hand to the epigastrium, and exercised strong pressure ; there was slight amelioration, the movements were less convulsive and the dyspnœa less intense. A large pad of linen was then applied over the epigastric region, and pressed strongly inward by means of a bandage passed around the body. In a very short time complete relief was obtained; the pad was left several hours in position, and when it was removed the symptoms did not return and have not since reappeared. —*Med. and Surg. Rep.*

**The Bacteria Fallacy Illustrated**.—Dr. Greg, Buffalo, N. Y., says that the three classified forms of so-called bacteria, in diphtheria, are nothing more than the three stages of the fibrillation of fibrin, of which the diphtheritic membranes are composed.

All the membranes of diphtheria are wholly, or almost wholly, composed of fibrin.

This fibrin is thrown out into the throat, or upon other parts, because it is in excess in the blood in this disease.

Thus it will be seen that this whole question of the membranes of diphtheria, the falsely assumed bacteria in connection therewith, the coagula of the heart in this disease, etc., may be placed at once upon a purely scientific basis, if the profession so desires. And by this showing, too, it will be seen that the exercise of a little common sense, and the proper application of a few simple facts to the solution of the subject, by the original promulgators and promoters of the bacteria theory, would have saved the medical profession a great disgrace, would have avoided hastening tens of thousands of patients out of the world in the vain effort to destroy by treatment what did not exist, as vegetable parasites, and would have rapidly advanced, instead of retarded, our knowledge of this terrible disease.

**To Pass the Esophageal Tube** sometimes is found very difficult, and dangerous delay may often be occasioned when the stomach pump is required in cases of poisoning. In such cases the attempt is generally made to pass the tube with the patient in the dorsal position, and its passage is frequently obstructed at some point in the esophagus. This annoying difficulty usually may be overcome by holding the patient in the upright position during the passage of the tube. We do not know the author of this procedure, but remember having seen it successfully carried out in Bellevue Hospital, when all attempts to pass the tube, with the patient in the dorsal position, had failed.—*Toledo Med. Journal.*

**Iodide of Potassium in Cardiac Dyspnœa.**—Iodide of potassium has been found by Professor See to work well in all cases of continuous cardia dyspnœa, particularly when this is connected with some structural lesion. It is equally useful in valvular lesions. No evil results can occur from its use, even if a mistake is made and the affection is asthmatic. The iodine liquifies the bronchial secretion. The dose is twenty grains a day, gradually increased to two or two and a half scruples. A good formula is:

℞ Potas. iod.......................................... ʒ vss

Syr. aurantii cort.................................. f. ℥ iv

Sig. Two to four teaspoonfuls a day in a tumbler of water.

Patients suffering from heart disease are more tolerant of iodide of potassium than other patients. The contra-indications to its use are: 1, tendency to hemorrhage; 2, loss of flesh; 3, loss of strength: 4, loss of appetite. Opium may be added to prevent iodism. Another combination is digitalis with iodine, as one has a soothing influence on the dyspnœa by acting on the lungs, and the other increases the action of the heart and modifies the arterial tension. The following formula will be found to answer well:

℞ Potas. iod.......................................... ʒ ss

Tinct. digitalis.................................. f. ℥ ss

Syr. acaciæ.......................................... f. ℥ iv

Sig. Dessertspoonful four times a day.

When digitalis is unsuitable, chloral may be substituted.—*Drug. Circular.*

**The Land Origin of the Yellow Fever.**—Dr. Manuel Da Gama Lobo has made an elaborate study of the causes of yellow fever, and has summed up his conclusions in a pamphlet just published in New York. With his microscopic researches is given a system of careful observations on conditions of temperature, moisture, barometric pressure, and direction of winds, during twenty-six years, in the city of Rio Janeiro. The author believes he has fixed the origin of yellow fever in two places, Havana and Vera Cruz, as its chief nests, and he attributes it definitely to the poison of the "opunsia Mexicana," a species of infusoria, of the family of "bacillarum," which is found particularly abundant in the swamps and waters near those cities. Plates showing this species as existing in its native element and in yellow fever victims accompany the descriptions. The case is not put forth as one of absolute proof, but as a theory supported by strong probabilities. The author is physician to the Emperor of Brazil, and is a pupil of the distinguished Professor Virchow.—*Miss. Medical Monthly.*

**Chloral in Dysentery.**—*Il Racogltatore Medico* says that Curci gives first a mild purgative, then chloral combined with chlorate of potash; afterwards, chloral alone in barley gruel, either by the mouth (one to three grams = 15 to 45 grains a day, for an adult), or by enema (19 grams = 154 grains, in two litres = 3½ pints of gruel, for 10 injections.

**Abortive Treatment of Small Pox by Salicylic Acid.**—Dr. Edwin Rosenthal, acting on the article by Dr. Boyer, has employed salicylic acid in many cases of small pox with good results. The formula employed by him is as follows :

| | |
|---|---|
| ℞ Ac di salicylici | 1 drachm. |
| Spts vini rectificati | ½ ounce. |

Mix and add :

| | |
|---|---|
| Elix. simplici, q. s | 6 ounces. |

For the angina of variola, he uses in conjunction therewith, the following gargle of xylol, and finds it very satisfactory :

| | |
|---|---|
| ℞ Xylol | 1 drachm. |
| Gum acaciæ | 2 " |
| Aq. menth. pip | 6 ounces. |

Misce, fiat emulcio.

Use as a gargle and mouth wash. He confirms the statement that salicylic acid in small pox reduces the temperature, is sedative, and modifies the eruption.—*Medical Bulletin.*

**Infantile Diarrhœa.**—For children belonging to families in easy circumstances, M. J. Guerin mixes a certain quantity of Belloc's powder of charcoal with each milk meal—half a teaspoonful at each meal. For the children of the working classes, Belloc's powder, which is a little dear, is replaced by very finely powdered, farina-like, ground baker's charcoal. This powder mixes readily with milk, and children drink the mixture as though the milk were pure. In a very short time, sometimes on the first day, the stools change in consistence and odor, and instead of being green, become blackish-yellow. At the same time that this addition is made, M. Guerin dilutes the milk with one-third or one-half of sweetened water, and the children take it without repugnance or vomiting. M. Guerin has frequently seen children, exhausted by seven or eight days' uncontrollable diarrhœa, regain in two or three days the expression of health.—*London Med. Record.*

**Lister's Antiseptic Treatment in Surgical Wounds.**—Dr. Boeckel, (Gaz. Med. de Strasbourg, December 1st, 1880), gives a table of statistics of major amputations performed by himself, some with and some without antiseptic precautions. Fifteen amputations of the thigh were performed antiseptically with four deaths, and seven were treated otherwise with three deaths ; eighteen amputations of the leg were treated antiseptically without a death, and nineteen treated in other ways with four deaths. In going into the causes of death, the author concludes that in neither case can the deaths be attributed to the method of dressing employed. Nevertheless, he thinks that the advantage is decidedly with the cases treated antiseptically, on account of the rapid healing, the absence of fever and of suppuration, and the rarity of the dressings in these cases. He mentions the occurrence of septic fever in a few cases, and, with Edelberg, he attributes this to absorption of blood from the wound.—*London Med. Record.*

**Double Irrigation and Injection Tube.**—Dr. H. O. Marcy, of Boston, Mass., exhibited at American Medical Association some soft rubber double tubes, which could be used as stomach, uterine or rectal tubes, and as catheters.

Dr. Gonley said he had a double soft rubber catheter for five years, but he never uses it in the male bladder. Irrigation should be done as quickly as possible, and not kept up constantly,

Dr. Marcy believed cystitis could be treated satisfactorily with heat, which could be applied by means of the catheter exhibited.

Dr. A. Byrd, of Quincy, Ill., uses a single rubber tube for a stomach tube, and fills it from a funnel, reversing the patient and allowing the fluid to run out. Dr. Hodgen, of St. Louis, taught him this.

Dr. Hunter McGuire mentioned a case in which a soft rubber catheter was passed completely into the bladder, from whence he removed it the next day with a lithotrite.—*Va. Med. Monthly.*

THE Boston Commercial Bulletin tells the following: "Dr. —— is an eminent physician of Philadelphia, and, like some others of his class, is somewhat brusque and overbearing in his manner. One morning he found among his office patients a gentleman who, after occupying exactly five minutes of the great man's time, took a ten dollar note from his pocket and inquired the amount of his fee. "Fifty dollars," said the impatient man. The patient demurred a little, whereupon the physician rudely remarked; "Well, what do you expect to pay? Give me what you have got." And on receiving the ten dollar note turned scornfully to his negro servant, and handing him the money remarked: "That is for you, Jim," but lost his temper still more when the patient coolly remarked: "I did not know before that you had a partner. Good morning, doctor."

**To Terminate the Chloroform Narcosis.**—A peculiar device is mentioned by Schirmer in the February number of the Centralblatt f. Augenheilkunde. He claims to have used it in his clinic for many years, and often succeeded in producing inspiratory movements when other means failed. He also employed it to induce rapid recovery; for instance, in strabismus operations, in order to test the result. The method consists in irritating the nasal mucous membrane. It has long been known, at least to physiologists, that the fifth nerve retains its sensibility longer than any other part in narcosis, and that reflexes may be induced through this nerve when other irritations fail. Shirmer uses simply a rolled piece of paper, which he turns in the nose. In dangerous cases he dips the paper into ammonia.—*Chicago Med. Review.*

**A Prominent** physician of this city who was taking a mixture of cascara sagrada and strychnia for constipation, discovered that the alkaloid was acting as an aphrodisiac; not being in need of such a remedy, he wrote a note to a neighboring druggist, in which he stated the case, and requested that the prescription be refilled, minus the strychnia. His messenger returned with the medicine and the following laconic reply: "Here's your cascara; for G—d's sake send me the strychnia!"—*Obstetric Gazette.*

**Rhus Aromatica.—A Case.**—Mr. H. was taken in October last with an irresistible desire to urinate every ten or fifteen minutes, passing only a few drops of high colored urine, followed by burning pain in the urethra. Complained of a "letting down sensation in the region of the bladder," after which a few drops of blood passed. Many times the urine was preceded by a flow of mucus and followed by considerable blood. The patient expressed himself as having, while urinating, a "desire to raise upon tip toe and pull hard on something." My experience with rhus aromatica in several severe cases, of urinary trouble, induced me to give it another trial. Not having the tincture on hand, we added twenty drops of the fluid extract to half a glass of water, and directed one teaspoonful every hour. In twenty-four hours all the severe symptoms were removed, and the patient made a good recovery.—*Med. Call.*

A SINGLE doctor like a sculler plies;
The patient lingers and by inches dies;
But two physicians, like a pair of oars,
Wafts him with swiftness to the Stygian shores.—*Ex.*

REJOINDER.

*Snooks*, happy, cheerful, stout and well,
Let all the Doctors go to h—,
Snooks, with cramp colic, on his knees—
Dear Doctor, come and give me ease!

**Jamaica Dogwood.**—In Brazil the Jamaica Dogwood has, it is said, (Therapeutic Gazette) an established reputation as a nervous sedative. Its action seems to be over the nervous centres; it causes sleep without producing the cerebral hyperæmia, nausea and nervous disturbances, which succeed opium and morphia. The sleep is tranquil and refreshing; it soothes bronchial coughs, and moderates the paroxysm of asthma and nervous coughs.

The active principle is a resinoid, soluble only in strong alcohol.

The dose of the fluid extract is from 30 drops to two fluid drachms. It is applied externally as well as given internally.

Its most valuable therapeutic use is assuaging nervous pain and producing sleep.

**Aconite in Remittent Fevers.**—Dr. Gerald Bomford, of Fort William, Calcutta, writes to the Practitioner (London): The good effects of aconite in this class of fevers may be summed up as follows:

1. It reduces the temperature.
2. It reduces the rapidity of the pulse, and makes it full and strong.
3. It cleans the tongue, and restores the digestive functions.
4. It induces sleep.
5. It increases the quantity of urine, and seems to have a direct effect in removing the symptomatic congestion of the kidneys.
6. It promotes perspiration. I may add that it is exceedingly grateful to the palate of a fever patient.—*Amer. Practitioner.*

**Diphtheria.**—Dr. J. R. Jones says, in Detroit Lancet: Of the various remedies now used and advised which I have tried, Monsel's solution of the subsulphate of iron has proved the most efficient for checking the spreading of the false membrane and causing its removal. Brushed on thoroughly with a camel's hair pencil, or used as recommended by Dr. J. Lewis Smith, in his latest edition of "Diseases of Children," it has, in my hands, proved eminently satisfactory. It does not burn nor irritate, but causes a disagreeable puckering of the throat, and although the patients do not like it, they readily submit to its application from the relief it affords.

**Treatment of Cholera Infantum.**—Soltman, in *Breslau Aerstl. Zeitschrift* uses resorcin, in doses of ten to thirty centigrams (1 2-3 to 5 grains), in 60 grams (two ounces) infusion of chamomile, for children a few months old. Its action is marked within two days, but a cure is obtained, on an average, in six days.

Resorcin, like carbolic acid, is an anti-ferment, but it is not irritating, and produces no symptoms of intoxication in medicinal doses, like the latter. Patients take it willingly and it is tolerated by the stomach; under its influence the digestive tube quickly recovers its function of assimilation which it had lost.—*Ex.*

**Cure of Goiter by Fluoric Acid.**—Dr. Edward Woakes gives, in the Lancet, a detailed account of a number of cases of goiter cured by fluoric acid internally. He begins treatment with fifteen minims of a one-half per cent. dilution of the acid three times a day, and, if necessary, increases the dose to twenty, thirty, forty, or even seventy minims, and extends the time to several months. His results are quite remarkable, even in cases that had resisted iodine, bromine, iron, etc. In a few it was conjoined with injections of tincture iodine. Very few failed to be reasonably benefited, and in eighty-five per cent. the cure was decided.—*Independent Practitioner.*

**Radical Cure of Rupture.**—The secret method of cure practiced by Dr. George Heaton successfully in one hundred and forty cases is now, after his death, published by Dr. J. H. Davenport. He injected extract of quercus alba into the hernial canal outside the peritoneal sac, to excite a mild degree of irritation in the tendons and fasciæ, so as to lead to contraction. No fatal results followed nor any serious complications. It often cured, and when it failed great relief was obtained, so that a light truss sufficed to support the protrusion. —*Independent Practitioner.*

**Ice to the Abdomen in Typhoid Fever.**—At a recent seance of the Societe Medicale des Hopitaux, M. L'abbe called attention to the efficacy of ice applications to the abdomen in typhoid fever, complicated or not. He related the case of a young girl attacked with typhoid, whose temperature exceeded 104°, and who appeared at the last extremity, who, under the influence of this treatment, was perfectly cured. M. L'abbe claims for this procedure a considerable lowering of the temperature and a notable amelioration of all the other symptoms.—*Canada Medical Record.*

